# Unmasking the Invisible: A Case Study of Aspiration Pneumonia Unveiling a Bronchoesophageal Fistula

**DOI:** 10.7759/cureus.59145

**Published:** 2024-04-27

**Authors:** Arman Sindhu, Ulhas Jadhav, Babaji Ghewade, Jay Bhanushali, Pallavi Yadav

**Affiliations:** 1 Respiratory Medicine, Jawaharlal Nehru Medical College, Datta Meghe Institute of Higher Education and Research, Wardha, IND; 2 Obstetrics and Gynecology, Jawaharlal Nehru Medical College, Datta Meghe Institute of Higher Education and Research, Wardha, IND

**Keywords:** bronchoesophageal fistula, diagnostic challenges, multidisciplinary approach, endoscopic intervention, squamous cell carcinoma, aspiration pneumonitis

## Abstract

Bronchoesophageal fistula (BEF) is a rare, yet clinically significant, condition characterized by an abnormal connection between the bronchial tree and the esophagus. We present the case of a 25-year-old female who initially presented with symptoms of aspiration pneumonitis and was subsequently diagnosed with BEF, attributed to poorly differentiated squamous cell carcinoma. Despite initial attempts at palliative intervention through esophageal stent placement, persistent symptoms prompted further investigation, revealing the underlying malignancy. This case underscores the diagnostic challenges associated with BEF, particularly when malignancy is involved, and emphasizes the importance of a multidisciplinary approach in optimizing patient outcomes. Early recognition, thorough evaluation, and comprehensive oncological management are essential in addressing the clinical complexities posed by BEF. Further research is warranted to better understand the pathophysiology and optimal management strategies for this rare but clinically significant condition.

## Introduction

Bronchoesophageal fistula (BEF) is a rare pathological condition characterized by an abnormal connection between the bronchial tree and the esophagus. It can result from various etiologies, including congenital malformations, trauma, inflammatory diseases, and malignancies [1]. Although BEF is uncommon, its clinical significance lies in its potential to cause severe respiratory complications such as recurrent pneumonia, aspiration pneumonitis, and respiratory distress, which can lead to significant morbidity and mortality if left untreated [2]. The clinical presentation of BEF often includes symptoms such as coughing, dyspnea, recurrent pneumonia, hemoptysis, and gastrointestinal complaints like dysphagia or regurgitation [3]. Diagnosis typically involves a combination of imaging modalities such as chest X-ray, CT scans, and endoscopic procedures like bronchoscopy and esophagoscopy. Confirmation of the diagnosis is crucial for appropriate management, including endoscopic interventions, surgical repair, or treatment of underlying conditions such as malignancy [4].

Endoscopic interventions such as stent placement have emerged as a viable option for the palliative management of BEF, offering symptom relief and improving quality of life in select cases [5]. However, definitive treatment often depends on addressing the underlying cause, especially when malignancy is involved. Multidisciplinary management involving gastroenterologists, pulmonologists, thoracic surgeons, and oncologists is essential for optimizing outcomes in patients with BEF [6]. Here, we present a case of aspiration pneumonitis secondary to BEF in a young female ultimately diagnosed with poorly differentiated squamous cell carcinoma. This case highlights the importance of considering BEF in the differential diagnosis of patients presenting with recurrent respiratory symptoms. It underscores the need for prompt diagnosis and multidisciplinary management for optimal outcomes.

## Case presentation

A 25-year-old female presented to the emergency department with a 15-day history of cough with expectoration, fever, and breathlessness on exertion. She also reported weight loss and decreased appetite over the same duration. Before this presentation, she had been admitted to a rural hospital with similar complaints but was later referred to our center for further management.

**Figure 1:**
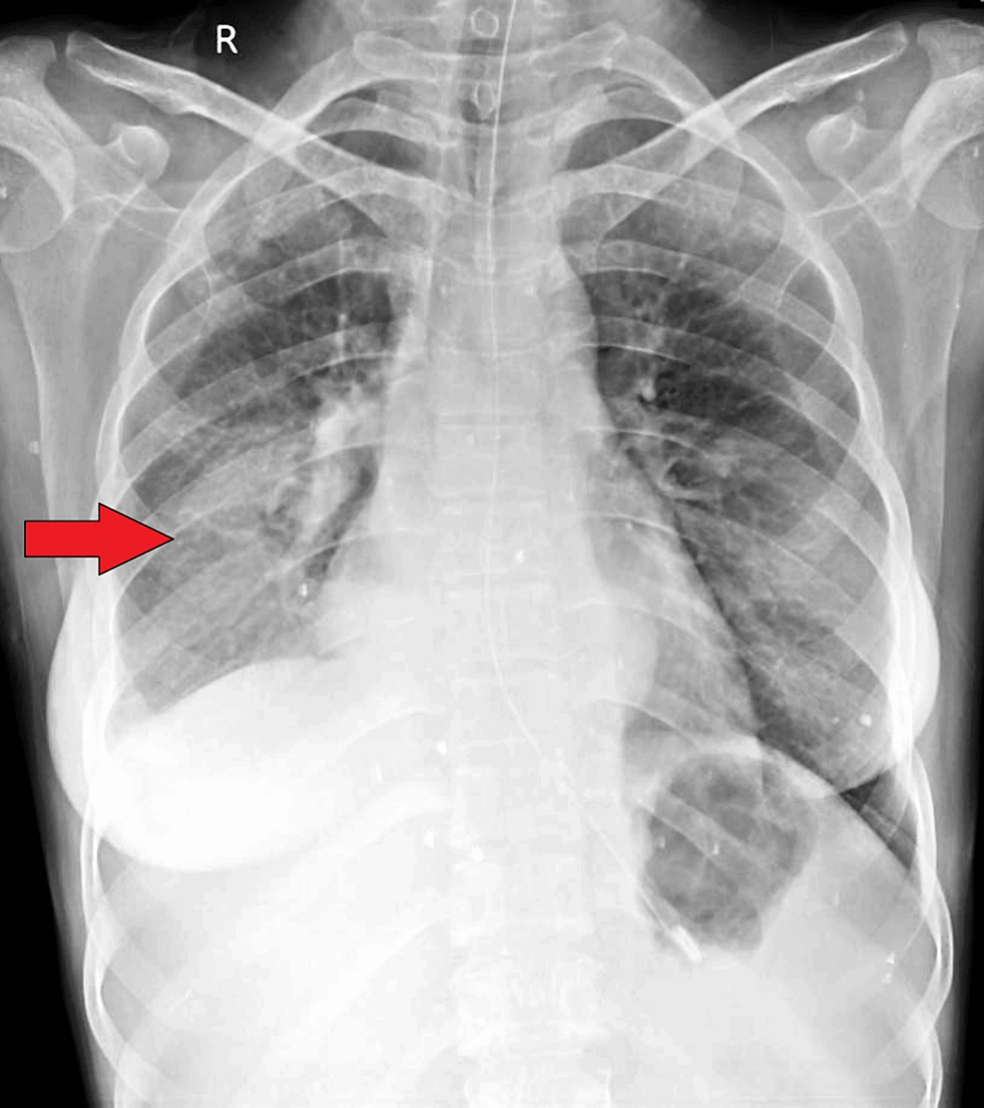
A chest X-ray done on admission showing homogenous opacity in the right lower lobe

The patient reported coughing episodes after ingesting water or semi-solid meals. There was no significant past medical history or history of trauma. On initial examination, she was lethargic with tachycardia and reduced breath sounds in the right mammary area on lung auscultation. Abdominal examination was unremarkable. A radiological examination of the chest X-ray on admission showed homogenous opacity in the right lower lobe (Figure 1).

**Figure 2:**
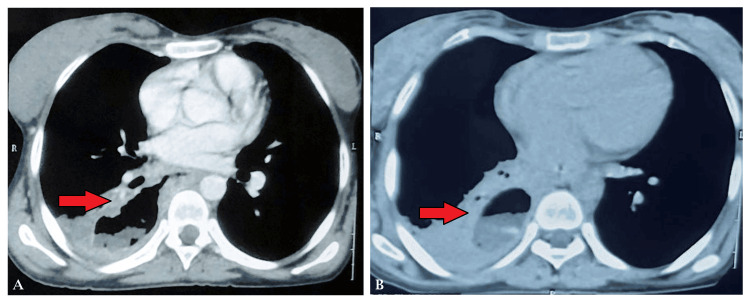
(A) An axial section of the CT of the thorax showing a communication between the esophagus and the right bronchus. (B) An axial section of the CT of the thorax showing a fluid-filled cavity in the right posterior segment of the lower lobe

Her initial vital signs were notable for a pulse of 138 beats per minute, a blood pressure of 110/70 mm Hg, a respiratory rate of 24 breaths per minute, an oxygen saturation of 96% on room air, and an oral temperature of 38°C. High-resolution CT of the thorax revealed a heterogeneous opacity in the right lower lobe with communication between the lower one-third of the esophagus and the lower lobe of the lung, consistent with a BEF (Figure 2A-2B).

**Figure 3:**
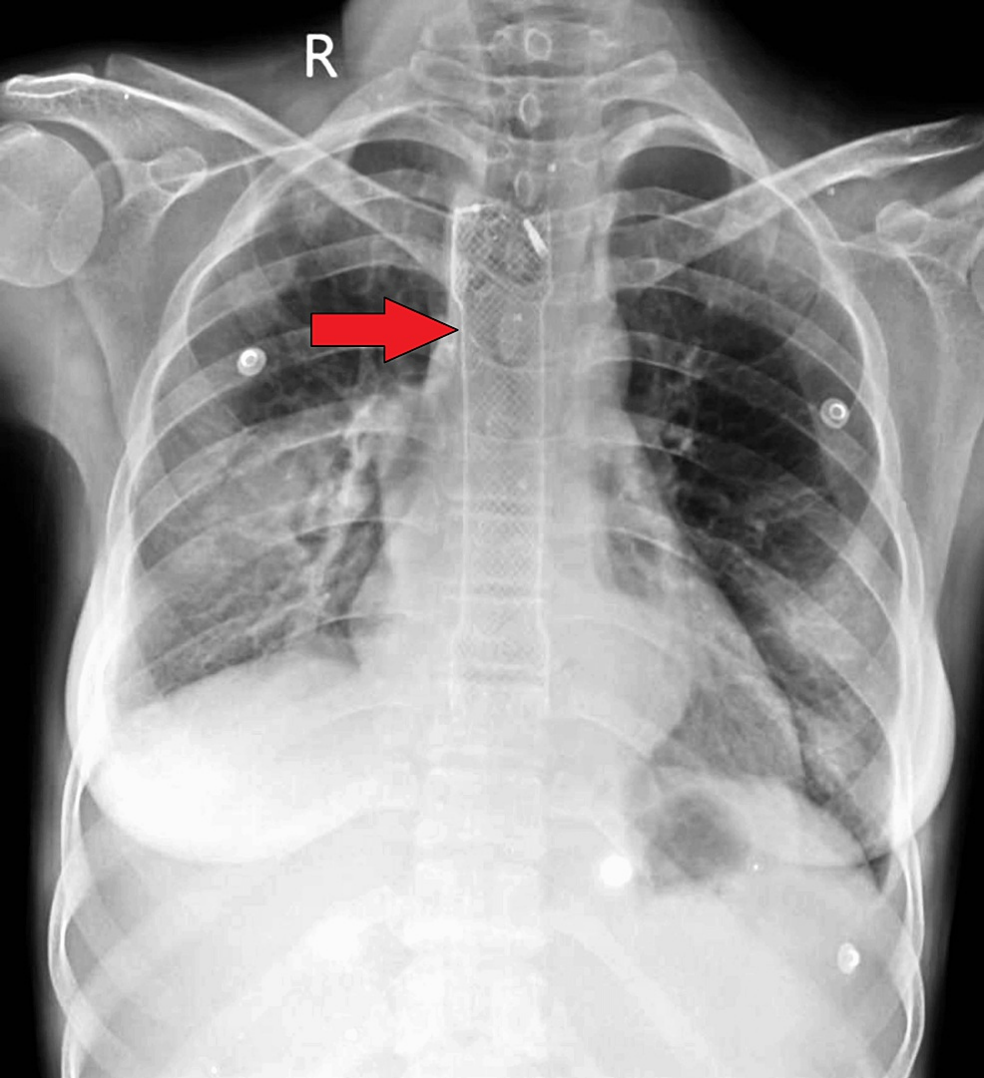
A chest X-ray posteroanterior view, showing the placement of the esophageal stent

**Figure 4:**
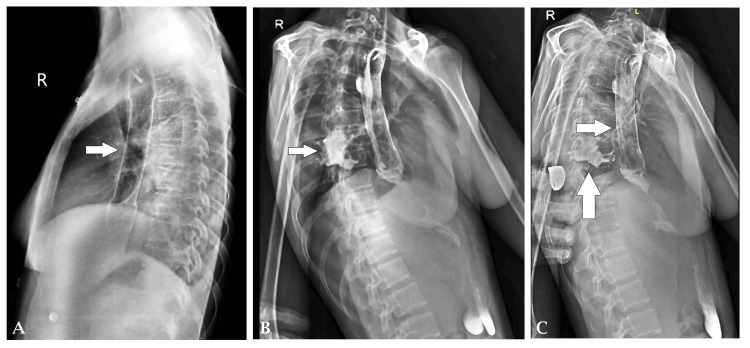
(A) A lateral chest X-ray showing the placement of the esophageal stent. (B) Contrast study showing leak of contrast from the esophagus to the right bronchus with a tract filled with contrast. (C) Contrast study showing leak of contrast in the cavity in the right lower lobe

Flexible optic bronchoscopy confirmed the fistulous connection between the segmental bronchus and the esophagus. Methylene blue injected into the right lower lobe showed a decreased return on suction, and subsequent aspiration from the nasogastric tube revealed food particles mixed with methylene blue, further confirming the diagnosis. The patient underwent an endoscopy, which revealed a fistulous opening in the esophagus, 30 cm from the incisor teeth. An esophageal-covered metal stent with hemoclip placement was performed to manage the fistula. However, the patient did not experience relief from symptoms post-stent placement (Figure 3). A contrast study demonstrated contrast-filled tracts between the esophagus and the right bronchus, confirming the persistence of the fistula (Figure 4A-4C).

**Figure 5:**
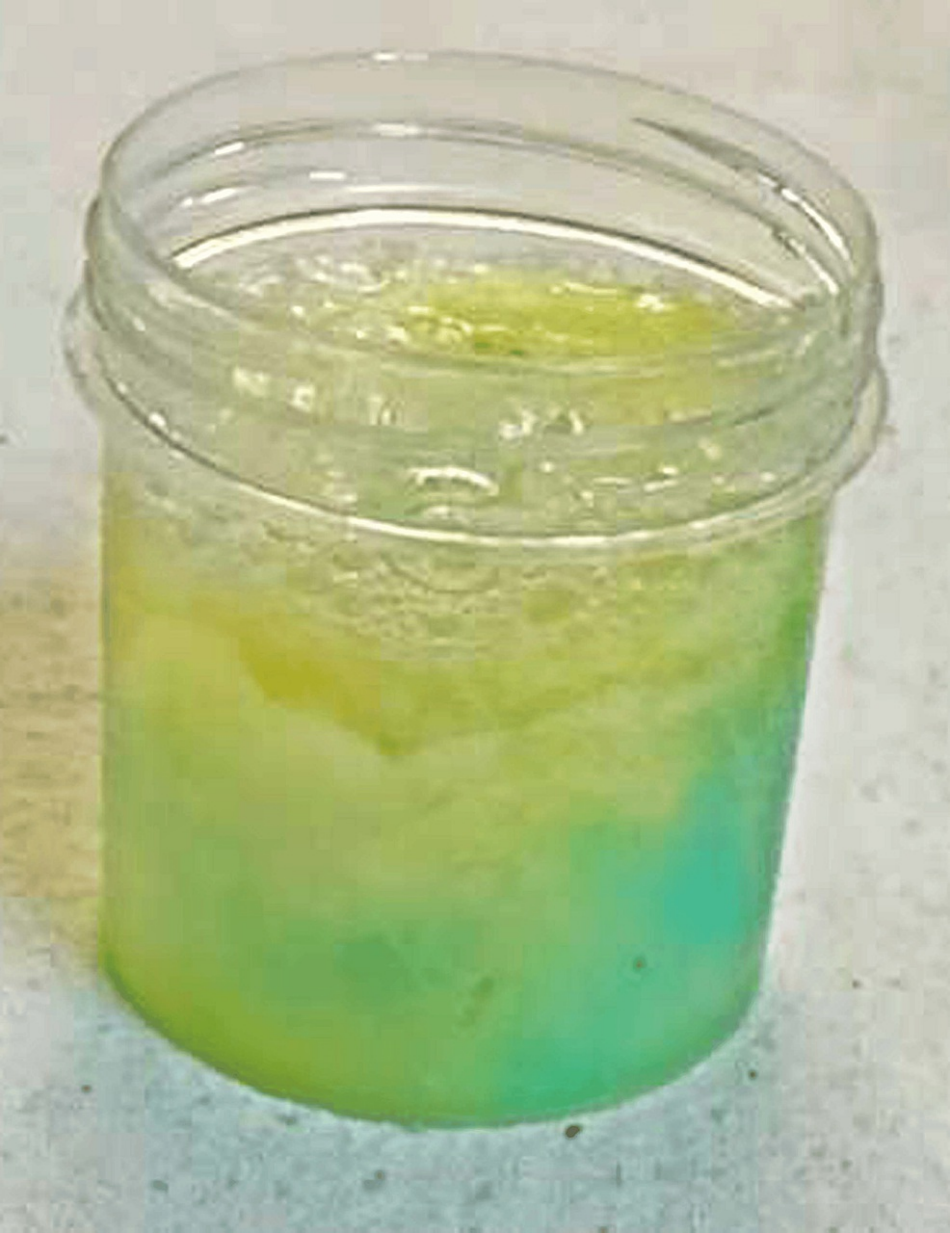
Nasogastric tube aspirate shows methylene blue injected during bronchoscopy in the right lower lobe suggesting a communication between the bronchus and the esophagus

Histopathological examination of biopsies taken during endoscopy revealed poorly differentiated squamous cell carcinoma underlying the fistula. Nasogastric tube aspirate shows methylene blue injected during bronchoscopy in the right lower lobe suggesting a communication between the bronchus and the esophagus (Figure 5). The patient was referred for further oncological management.

## Discussion

BEF is a rare yet clinically significant condition requiring prompt recognition and appropriate management. In this case, the patient presented with symptoms consistent with aspiration pneumonitis, which led to the discovery of a BEF ultimately attributed to poorly differentiated squamous cell carcinoma. BEF can arise from various etiologies, including malignancy, inflammation, trauma, or iatrogenic causes [7]. Malignancy is a well-recognized cause of BEF, with squamous cell carcinoma being the most common histological type associated with this condition [8]. Our case adds to the existing literature by highlighting the diagnostic challenges posed by BEF and emphasizing the importance of considering underlying malignancy, particularly in cases where symptoms persist despite initial interventions.

Endoscopic evaluation, including flexible bronchoscopy and esophagoscopy, is crucial in diagnosing BEF [9]. In our case, bronchoscopy revealed a fistulous connection between the bronchus and esophagus, further confirmed by contrast studies and histopathological examination. These findings align with previous studies demonstrating the utility of endoscopic modalities in diagnosing BEF and guiding subsequent management [10]. Management of BEF often involves a multidisciplinary approach, with interventions aimed at both symptom palliation and addressing the underlying cause [11]. In our case, placement of an esophageal stent was initially attempted to alleviate symptoms; however, the persistence of symptoms prompted further investigation, leading to the diagnosis of squamous cell carcinoma. This underscores the importance of thorough evaluation and consideration of underlying malignancy in patients with suspected BEF, as palliative measures alone may not provide definitive relief.

The prognosis of BEF depends largely on the underlying etiology and the stage of the disease at presentation. While benign causes of BEF may respond well to conservative management or endoscopic interventions, malignant BEF often carries a poorer prognosis and requires comprehensive oncological treatment [12]. In our case, the presence of squamous cell carcinoma necessitated further oncological evaluation and treatment planning.

## Conclusions

This case underscores the diagnostic challenges and complexities associated with BEF, particularly when malignancy is involved. Despite initial attempts at palliative intervention through esophageal stent placement, the persistence of symptoms prompted further investigation, ultimately leading to the diagnosis of poorly differentiated squamous cell carcinoma. This highlights the importance of considering underlying malignancy in patients with suspected BEF and emphasizes the need for a multidisciplinary approach to optimize patient outcomes. Early recognition, thorough evaluation, and comprehensive oncological management remain paramount in addressing the clinical complexities posed by BEF. Further research and clinical studies are warranted to better understand the pathophysiology and optimal management strategies for this rare but clinically significant condition.
